# Complete Genome Sequences of Two Bovine Viral Diarrhea Viruses Isolated from Brain Tissues of Nonambulatory (Downer) Cattle

**DOI:** 10.1128/genomeA.00733-13

**Published:** 2013-09-12

**Authors:** Jae-Ku Oem, Soo-Kyung Joo, Dong-Jun An

**Affiliations:** Animal and Plant Quarantine Agency, Anyang si, Gyeonggi do, Republic of Korea

## Abstract

Here, we report the complete genome sequences of two bovine viral diarrhea viruses (BVDVs) (strains 11F011 and 12F004) isolated from brain tissues from nonambulatory (downer) cattle. The complete genomes of strains 11F011 and 12F004 contain 12,287 nucleotides (nt) with a single large open reading frame and 12,301 nt with a single large open reading frame, respectively. Phylogenetic analysis indicated that these strains belong to the BVDV-2a and -1b genotypes, respectively.

## GENOME ANNOUNCEMENT

Bovine viral diarrhea virus (BVDV) is an important pathogen that infects cattle, causing a number enteric, respiratory, and acute/chronic mucosal diseases and/or immunosuppression or immunotolerance, congenital defects, reproductive failure, and subclinical or persistent infections (1). Little is known about the mechanisms underlying the neuropathology and neurovirulence of BVDVs in cattle, although several reports have examined the neuropathogenesis and distribution of BVDV antigens within the central nervous system (CNS) (2–6). The most common brain lesion observed in calves transplacentally infected with BVDV was cerebellar hypoplasia, which is often associated with hydranencephaly, internal hydrocephalus, microencephaly, or porencephaly (5). Another study reported that all calves examined suffered from severe diffuse neuraxial hypomyelination; immunohistochemical analyses showed cerebral neuronal staining patterns consistent with congenital persistent *Pestivirus* infection (7).

Here, we report the complete genomic sequences of two novel BVDV strains, 11F011 and 12F004, which were isolated from brain tissues obtained from nonambulatory (downer) cattle in South Korea in 2011 and 2012, respectively. Nonambulatory cattle (commonly referred to as “downer”) are unable to stand up or walk. Total viral RNA was extracted from infected Madin-Darby bovine kidney epithelial (MDBK) cells using an RNeasy mini kit (catalog no. 74104; Qiagen). cDNA was obtained using a OneStep reverse transcription (RT)-PCR kit (catalog no. 210210; Qiagen). Ten sets of primers were designed based on conserved sequences identified from other BVDVs (accession no. M96751, U63479, M96687, U18059, and AF002227) from the GenBank database at NCBI. The PCR amplicons were cloned into the pGEM-T plasmid and then sequenced using universal primers (M13F and M13R) and an ABI Prism 3730xl DNA sequencer at the Cosmo Genetech Institute (Cosmo Genetech Co., Ltd.). All fragments were sequenced in both directions and the sequences were aligned using ClustalX 1.83 (8). A phylogenetic tree was then constructed in Mega 4.1 using the neighbor-joining method.

The complete genome of strain 11F011 comprises 12,287 nucleotides (nt), including a 386-nt 5′ untranslated region (UTR) and a 210-nt 3′ UTR. The complete genome of strain 12F004 comprises 12,301 nt, including a 379-nt 5′ UTR and a 228-nt 3′ UTR. The open reading frames of 11F011 and 12F004 encode polyproteins of 3,897 amino acids (aa) and 3,898 aa, respectively. The structural proteins of each strain contain 13 potential *N*-linked glycosylation sites.

A similar analysis of 30 complete BVDV genome sequences deposited in GenBank revealed that 11F011 shows 97% nucleotide sequence homology with strain P11Q and that 12F004 shows 93% nucleotide sequence homology with strain CP7. Phylogenetic analysis indicated that strains 11F011 and 12F004 belong to the BVDV-2a and -1b genotypes, respectively.

This is the first study to report the complete genome sequences of two BVDV strains isolated from brain tissues obtained from nonambulatory (downer) cattle. These sequences will form the basis for further studies to examine the molecular characteristics of the viruses. Such studies may help to identify the mechanisms underlying the neurologic sequelae associated with BVDV.

### Nucleotide sequence accession numbers.

The complete genome sequences of two novel BVDV strains, 12F004 and 11F011, were deposited in GenBank under accession no. KC963967 and KC963968.
